# Computed tomography manifestations of common inferior vena cava dysplasia and its clinical significance

**DOI:** 10.3892/etm.2012.830

**Published:** 2012-11-23

**Authors:** ZHEN-YU QIAN, MING-FENG YANG, KE-QIANG ZUO, JIE CHENG, HONG-BING XIAO, WEI-XING DING

**Affiliations:** 1 Department of General Surgery, Tongji Hospital, Tongji University, Shanghai 200065;; 2 Department of Surgery, Dongyang People’s Hospital, Dongyang, Zhejiang 322103;; 3 Department of General Surgery, Shanghai 10th People’s Hospital, Tongji University School of Medicine, Shanghai 200072, P.R. China

**Keywords:** anomaly, inferior vena cava, embryogenesis, cross-sectional imaging, computed tomography, posterior nutcracker phenomenon

## Abstract

This study aimed to review and analyse the computed tomography (CT) imaging results of frequently encountered developmental anomalies of the inferior vena cava (IVC). The underlying clinical significance was evaluated with reference to the relevant literature. CT images of patients who received abdominal or thoracic scanning between July 2009 and September 2011 were reviewed. Developmental anomalies observed in the IVC were identified and categorised. Images of the cases with typical anomalies were presented and their developmental mechanism, as well as clinical significance, was discussed. The most frequently encountered IVC developmental anomalies include the left vena cava, double vena cava, azygos continuation of the IVC, left circumaortic renal vein, left retroaortic renal vein and retrocaval ureter. The embryogenesis of the IVC is a complex process that results in various congenital anomalies. The developmental anomalies of the IVC are distinguished using a CT scan and have significant implications on clinical perspective.

## Introduction

Developmental anomalies of the inferior vena cava (IVC) include various congenital malformations that are traced to the embryogenesis of the IVC. Since the earliest description by Abernethy and Banks (1) in 1793, these anomalies have been regarded as normal variations of the retroperitoneal venous system for over a century, since they rarely occur and are mainly distinguished in cadaver dissection or accidentally during surgical exploration. Moreover, these anomalies cause no significant lifelong problems due to their latency (2). Thus, the clinical significance of these conditions has long been underestimated. Advances in medical imaging technologies and the increasing availability and accessibility of these methods in clinical use in the recent decades have provided a reliable method to investigate these anomalies. Various types of the anomalies have been identified with the help of cross-sectional imaging (3). Imaging results also serve as a valuable tool for clinicians to re-evaluate several clinical conditions, including haematuria (4) and urinary tract infection (5) of unknown aetiology, as well as recurrent deep vein thrombosis (DVT) and pulmonary embolism (6,7), which used to be refractory to conventional treatment and is currently considered to be related to the underlying anomalies of the IVC.

Despite these advances, knowledge of these anomalies remains insufficient among most radiologists and clinicians, which may lead to misdiagnosis in certain circumstances (8). For an interventional radiologist or surgeon, overlooking the existence of IVC anomalies may pose potential procedural risk. Hence, a better understanding of these anomalies and familiarity with their imaging features has become an essential task for radiologists and clinicians, to reduce misinterpretation and achieve accurate diagnosis. Cases of several typical developmental IVC anomalies diagnosed by an abdominal CT scan are reported in this study and the underlying clinical significance was evaluated by reviewing relevant literature.

Although the incidence rate of IVC dysplasia is significant, this condition generally has no serious consequences. IVC dysplagia is occasionally detected during an imaging examination or when surgery on a related area is conducted. The condition commonly includes left IVC, double IVC, IVC absence in the hepatic segment, left renal vein around the aorta, retroaortic left renal vein and retrocaval ureter (1). The misinterpretation of this condition as another lesion may be avoided by providing important clinical information. In the present study, computed tomography (CT) manifestations of six different types of IVC dysplasia cases are reported and their clinical significance is discussed.

## Materials and methods

### General data

Several patients who received abdominal and chest CT examinations between July 2009 and September 2011 in the Dongyang People’s Hospital were reviewed. A number of cases presented IVC dysplasia. In addition, several cases could not be defined due to the insufficiency or non-enhancement of the examination range. Among these cases, six cases of various common IVC dysplasia with typical manifestations were reviewed as follows: 5 male cases and 1 female case, with ages ranging from 43 to 73 years. This study was conducted in accordance with the declaration of Helsinki. This study was conducted with approval from the Ethics Committee of Tongji University School of Medicine. Written informed consent was obtained from all participants.

### CT examination method and image processing

Philips Brilliance 64-row spiral CT was used. According to the corresponding examination items, enhanced or plain CT scanning examination of the abdomen or chest was conducted, with a scanning layer thickness of 5 mm. The examination results were entered into the picture archiving and communication systems and saved for reading.

## Results

A total of six common IVC dysplasia cases were identified and six different typical cases were reported.

### Case 1: left IVC

**Figure 1.**
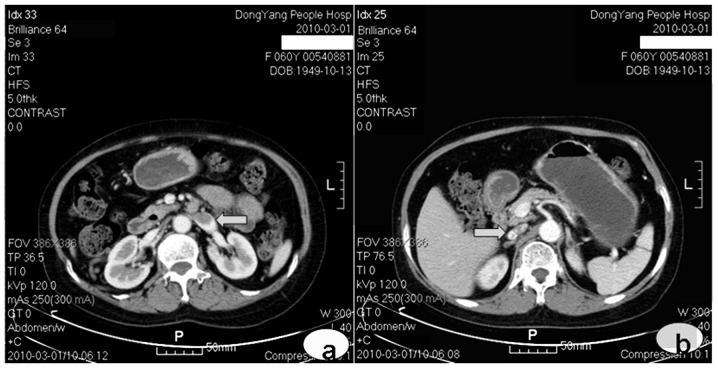
Arterial phase: (a) the left inferior vena cava (IVC) at the left renal vein abouchement level (arrow) and (b) the IVC running normally over the right renal vein level (arrow).

The patient was female and aged 60 years. Abdominal enhanced CT was conducted for re-examination following chemotherapy, due to non-Hodgkin lymphoma. The left renal vein extended out a large thick vascular shadow downward and the enhancement conditions complied with the veins. The condition was also accompanied by an abdominal aorta to the left side of the normal position of the abdominal aorta, which branched into the bilateral common iliac veins at the lumbar 5 vertebral level. Over the right renal vein level, its continuous large thick vascular shadow was invisible. Over the right renal vein level, it extended out as a normal running IVC (Fig. 1). This abnormality was diagnosed as left IVC.

### Case 2: double IVC

**Figure 2.**
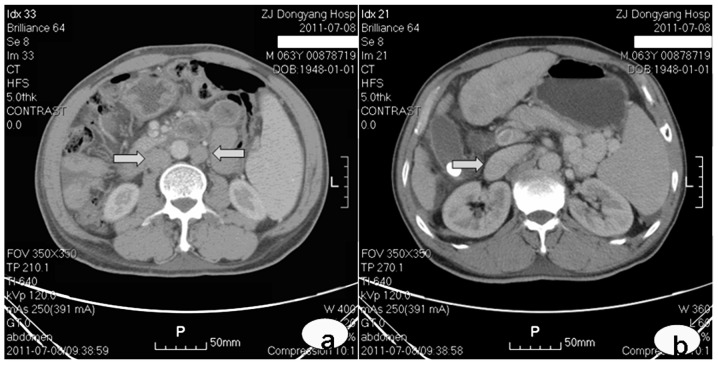
Venous phase: (a) the inferior vena cava (IVC) was visible on the left (as shown by the arrow pointing to the left) and right (as shown by the arrow pointing to the right) below the renal vein abouchement level and (b) the IVC running over the right renal vein level (arrow).

Abdominal enhanced CT was conducted in a 63-year-old male due to the space-occupying location in the hepatic left lobe and the presence of cholecystolithiasis, as confirmed by an ultrasound examination. The bilateral renal veins extended out a large thick vascular shadow downward. They were accompanied by the downward abdominal aorta at the bilateral sides of the abdominal aorta, which branched into the iliac veins at the lumbar 5 vertebral level. Over the right renal vein level, the bilateral renal veins extended out as a normal running IVC (Fig. 2). This condition was diagnosed as double IVC.

### Case 3: absence of IVC in the hepatic segment

**Figure 3.**
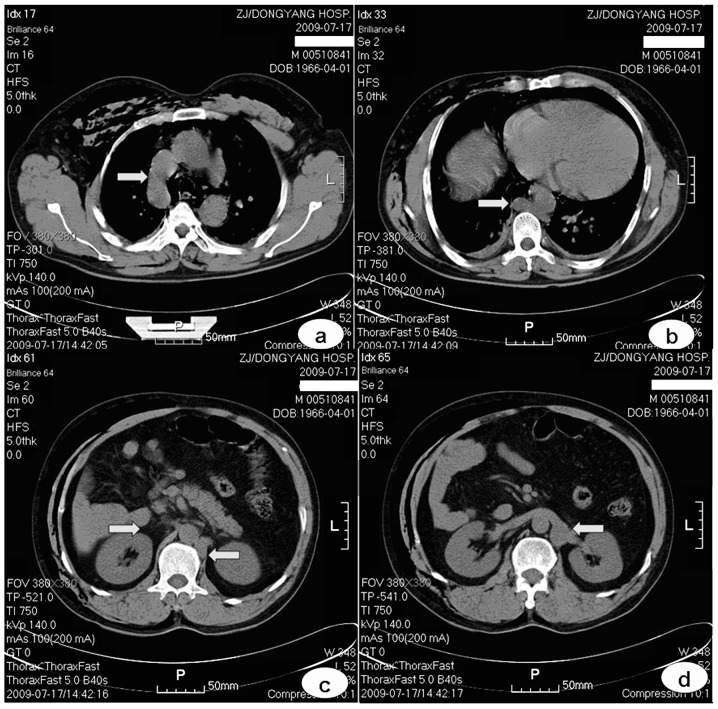
Plain scanning: (a) the clearly-thickened azygos vein (arrow) arches converged into the superior vena cava; (b) the azygos vein continued left as the thickened hemiazygos vein (arrow) posterior to the aorta rear at the level of the 10th thoracic vertebra; (c) there was no inferior vena cava (IVC) over the right renal vein level (indicated by the arrow pointing to the right) and the hemiazygos vein (as shown by the arrow pointing to the left) ran posterior to the left crus of the diaphragm; (d) the thickened hemiazygos vein (arrow) at the renal vein level.

Pectoral and epigastric plain CT was conducted in a 43-year-old male due to an injured abdomen caused by a traffic accident. The azygos vein thickened (Fig. 3a) and continued as thickened hemiazygos vein to the pectoral vertebral level 10 body (Fig. 3b) and ran downward to the left renal vein behind the left crus of the diaphragm (Figs. 3c and d). No IVC was visible over the right renal vein level. Therefore, the absence of IVC in the hepatic segment was considered. Left IVC was possibly present; however, this cannot be confirmed since hypogastric scanning was not performed. This case of CT examination showed no enhancement; however, vessel running is clearly shown compared with the perivascular adipose tissue with trauma-induced mediastinal emphysema.

### Case 4: left renal vein around the aorta

**Figure 4.**
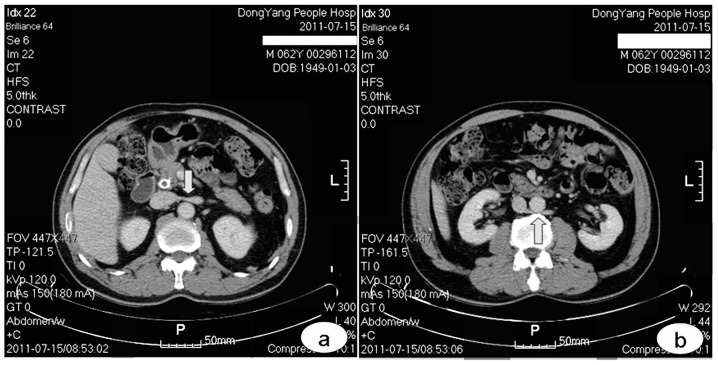
Venous phase: (a) the superior left renal vein (arrow) crossing in front of the abdominal aorta and (b) the inferior left renal vein (arrow) crossing behind the abdominal aorta.

A 62-year-old male underwent abdominal enhanced CT for re-examination after biliary stent implantation was conducted due to bile duct carcinoma. Aside from the superior left renal vein running anterior to the abdominal aorta (Fig. 4a), an additional inferior vessel draining the left kidney and crossing the abdominal aorta was observed to converge into the rear IVC (Fig. 4b). This abnormality was diagnosed as left renal vein around the aorta.

### Case 5: retroaortic left renal vein

**Figure 5.**
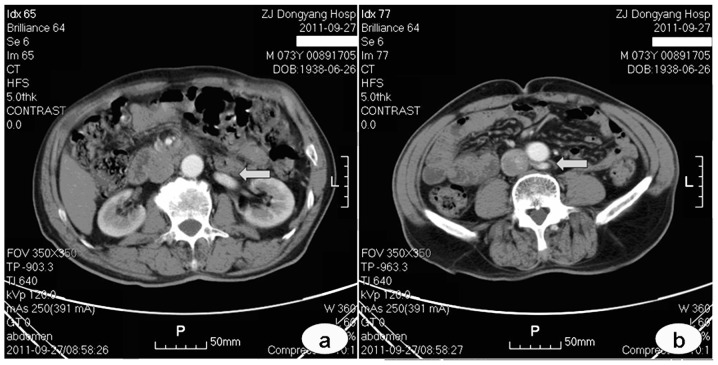
Arterial phase: (a) the running left renal vein (arrow) and (b) the left renal vein (arrow) crossing behind the abdominal aorta to converge into the subdermal space at a lumbar vertebra.

The patient was male and aged 73 years. Abdominal enhanced CT was conducted for re-examination following chemotherapy due to gastric cancer. The left renal vein ran downward to the right, crossing behind the abdominal aorta at the superior border of a lumbar vertebra to converge dorsally into the IVC (Fig. 5). This condition was diagnosed as retroaortic left renal vein.

### Case 6: retrocaval ureter

**Figure 6.**
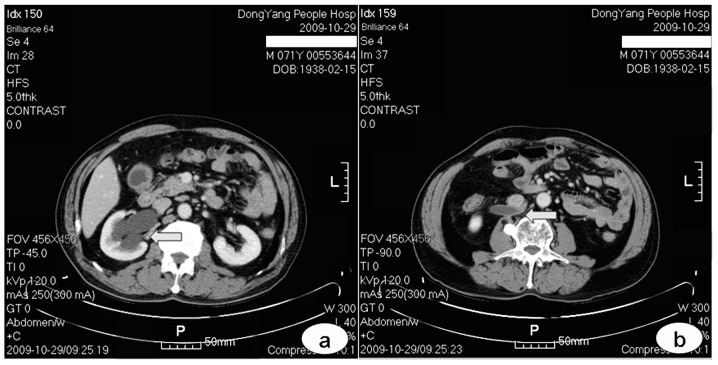
Venous phase: (a) the left hydronephrosis (arrow) and (b) the right ureter crossed behind the inferior vena cava (IVC). Hydronephrosis proximal to the crossing can be seen due to the obstruction of the ureter at this site.

A 71-year-old male underwent abdominal enhanced CT due to cecum carcinoma, as confirmed by enteroscopy. The right hydronephrosis was visible, renal parenchymal thickness was normal (Fig. 6a) and the right ureter abnormally ran inward and crossed behind the IVC (Fig. 6b). Hydrops were observed at the superior part and the inferior ureter had no apparent hydrops (Fig. 6b). This disease was diagnosed as retrocaval ureter.

## Discussion

The incidence rate of IVC dysplasia is significant. The embryonic development of this condition is complex and derived from the development of three pairs of embryonic veins, including the *vena postcardinalis*, subcardinal and supracardinal vein. Anastomosis, atrophy and disorders that occur during development will cause dysplasia (9).

A normal IVC includes four segments: hepatic, renal superior, renal and renal inferior segments (2). The hepatic segment is derived from the vitelline vein. The right subcardinal vein develops into the renal superior segment by forming subcardinal vein-hepatic segment anastomosis. The renal segment is derived from the right supracardinal vein-subcardinal vein and *vena postcardinalis*-subcardinal vein anastomoses. In the chest, the supracardinal vein develops into azygos and hemiazygos vein/accessory hemiazygos veins. In the abdomen, *vena postcardinalis* is gradually replaced by the subcardinal and supracardinal veins; however, it persists in existence and develops into the iliac vein in the pelvic cavity. Anastomosis between the dorsal supracardinal veins, between the ventral subcardinal veins and between the *vena postcardinalis*subcardinal veins constitute the renal collar. The embryonic kidney is first drained by pairs of ventral and dorsal branches. Bilateral dorsal branches degrade. On the right, the ventral branch integrates into the lateral wall of the IVC in the renal segment. On the left, the ventral and the anterior branches of the renal collar constitute the normal left renal vein. The embryonic ureter develops from the pelvic cavity after the posterior kidney develops and runs at the *vena postcardinalis* rear and at the anterior internal supracardinal vein. *Vena postcardinalis*-supracardinal vein anastomosis formed at the inferior part and supracardinal vein-subcardinal vein anastomosis formed on the renal level constitute a vein ring around the ureter. In a normal developmental process, the right *vena postcardinalis* degrades and withers, whereas the right supra-cardinal vein develops into the renal inferior segment IVC.

If the right supracardinal vein cannot form the normal right IVC due to an abnormal degeneration and the left supracardinal vein develops into an IVC in the renal inferior segment, the abnormality is diagnosed as left IVC (10). If both bilateral supracardinal veins develop into an IVC of the renal inferior segment, the condition is diagnosed as a double IVC (11). If subcardinal vein-hepatic segment anastomosis fails, continuous IVC are not developed. As a result, IVC from over the renal vein to the hepatic vein abouchement level is absent and hepatic veins converge to flow directly into the right atrium. This condition is known as the absence of IVC in the hepatic segment. Lower venous blood flow in the body flows back via the superior vena cava by the expanded azygos vein or hemiazygos vein (12). If the dorsal branch of the embryonic left renal vein and the posterior of the renal collar persist, two left renal veins are present; the left adrenal gland flows back via the superior left renal vein and the left gonadal vein flows back via the inferior left renal vein (13). If the ventral branch of the embryonic left renal vein and the anterior branch of the renal collar abnormally degrade and disappear, whereas the dorsal branch of the embryonic left renal vein and the posterior branch of the renal collar develop into the left renal vein, the abnormality is diagnosed as retroaortic left renal vein (14). If the right *vena postcardinalis* does not degrade, but develops into the main component of the IVC in the renal inferior segment, the right ureter that initially courses behind the IVC runs down inwardly to the inner side of the vein, and continues downward and finally circumvents anterior to it. Therefore, a retrocaval ureter forms (15). In addition, multiple types of common IVC dysplasia jointly form the complex cases (16).

Considering that a retrocaval ureter directly causes a serious left ureteral obstruction (17), more studies on this type of IVC dysplasia are available. In addition, other types of IVC dysplasia pose important clinical significance. Given the significant incidence of this condition, a better and complete interpretation of images is beneficial. Avoiding the misinterpretation of this condition as retroperitoneal lymph-adenopathy (18), urinary system abnormality, bossing or as another vessel is advantageous for understanding the various types of common IVC dysplasia and to master their imaging manifestations, particularly by CT and magnetic resonance imaging cross-sectional images. Although vasography confirms IVC dysplasia, this method is generally not considered as a diagnostic means. Surgeries via the IVC, including a variety of cardiovascular interventions and implantation of an IVC filter, require surgeons to be familiar with a variety of developmental anomalies. For vascular and general surgeons, as well as urologists, who are involved in the retroperitoneal region, identifying these conditions is necessary to prepare the corresponding surgery plan prior to surgery to prevent intra-operative misjudgement. In addition, IVC dysplasia changes the normal blood backflow route and possibly causes DVT (19). As the aorta stresses the left renal vein towards the lumbar vertebra, the retroaortic left renal vein may cause a left renal vein backflow disorder to induce haematuria, namely the posterior nutcracker syndrome (20).
